# Herpetic Meningoencephalitis Complicating the Resection of a Vestibular Schwannoma: A Case Report

**DOI:** 10.1002/ccr3.70050

**Published:** 2025-01-02

**Authors:** Jérôme Houdu, Maxime Barron, Thierry Civit, Cécile Parietti‐Winkler

**Affiliations:** ^1^ Department of Otorhinolaryngology Centre Hospitalier Régional Universitaire de Nancy, Hôpitaux de Brabois Vandœuvre‐Lès‐Nancy France; ^2^ Department of Neurosurgery Centre Hospitalier Régional Universitaire de Nancy, Hôpital Central Nancy France

**Keywords:** acoustic neuroma, case report, herpes simplex virus, herpetic encephalitis, vestibular schwannoma

## Abstract

After surgery involving cranial nerves and more generally the central nervous system, nonbacterial meningitis should raise suspicion of herpes simplex virus type 1 reactivation. No time should be wasted in diagnosis and treatment; therefore, a polymerase chain reaction testing on cerebrospinal fluid should be systematic in this situation, without neglecting to consider other differential diagnoses.

## Graphical abstract

A 58‐year‐old woman developed HSV‐1 meningoencephalitis a few days after resection of a vestibular schwannoma, diagnosed through polymerase chain reaction on cerebrospinal fluid, but initially suspected due to headaches, fever, and absence of bacteria in the cerebrospinal fluid. After surgery involving cranial nerves, non‐bacterial meningitis should raise suspicion of herpes simplex virus type 1 (HSV‐1) reactivation leading to herpes simplex meningitis. No time should be wasted in diagnosis and treatment; therefore, a PCR testing on CSF should be systematic in this situation, without neglecting to consider other differential diagnoses.

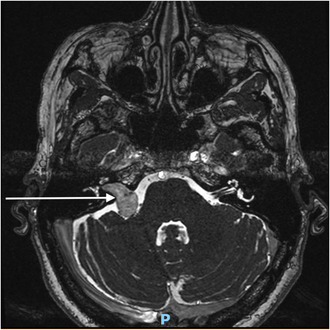

## 1 Introduction

Herpes simplex virus type 1 (HSV‐1) meningoencephalitis (HSVE) is the most common cause of sporadic encephalitis worldwide, although it remains rare: 2 to 4 cases per 1,000,000 [1]. The most common symptoms include confusion, behavioral disturbances, fever, seizures, headaches, and focal neurological deficits [1]. Polymerase chain reaction (PCR) in cerebrospinal fluid (CSF) is the gold standard test for its diagnosis, with 96% sensitivity and 99% specificity [2]. Given the high mortality rate (70% without treatment and 10% with treatment) and potential for sequelae [3], early diagnosis and treatment are crucial. There are no identified risk factors for HSVE, although fatigue, anxiety, and immunosuppression are suspected [4]. Fewer than 10 cases of HSVE following resection of a vestibular schwannoma have been reported in the literature [5, 6, 7, 8]. We want to draw the reader's attention to a diagnosis that could be overlooked in a postoperative context.

## 2 Case History

**FIGURE 1:**
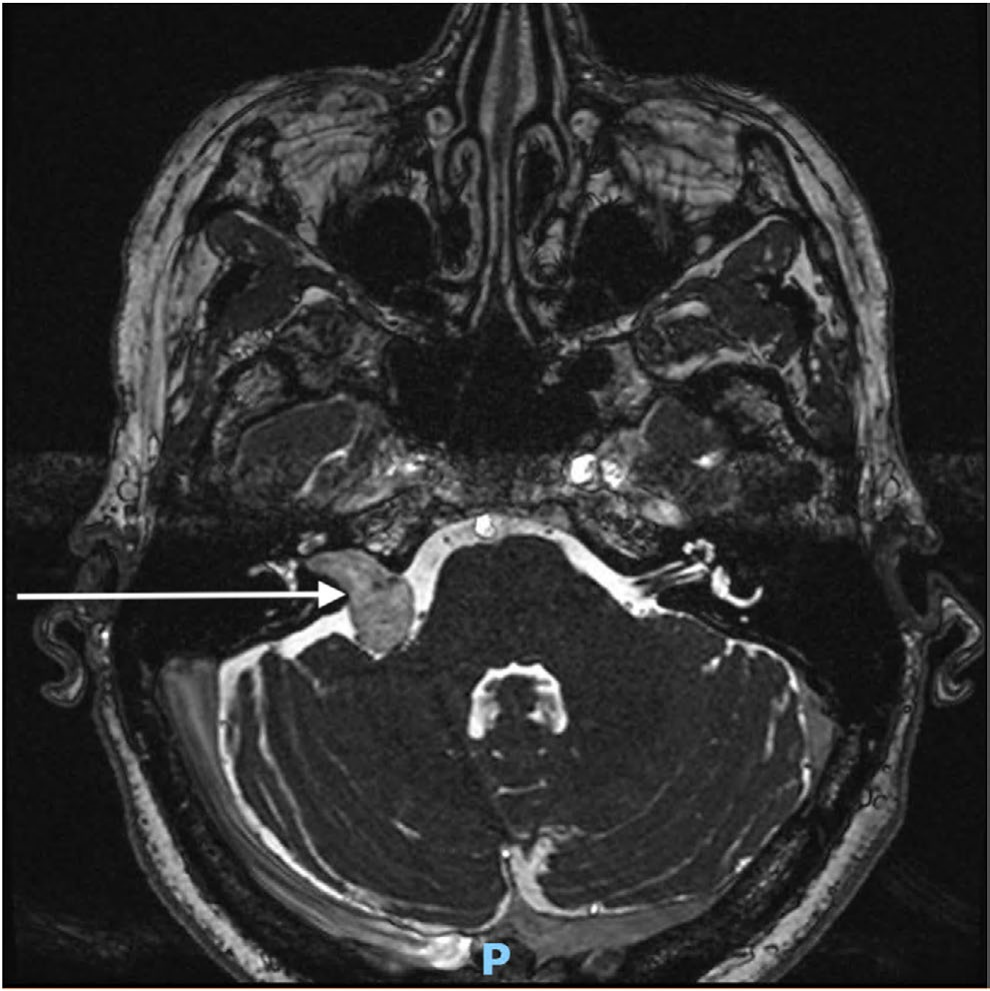
Preoperative MRI (T2‐weighted sequence) showing the vestibular schwannoma (white arrow) in the cerebellopontine angle. This tumor was classified as Koos III due to its close contact with the brainstem but without compression of it.

**FIGURE 2:**
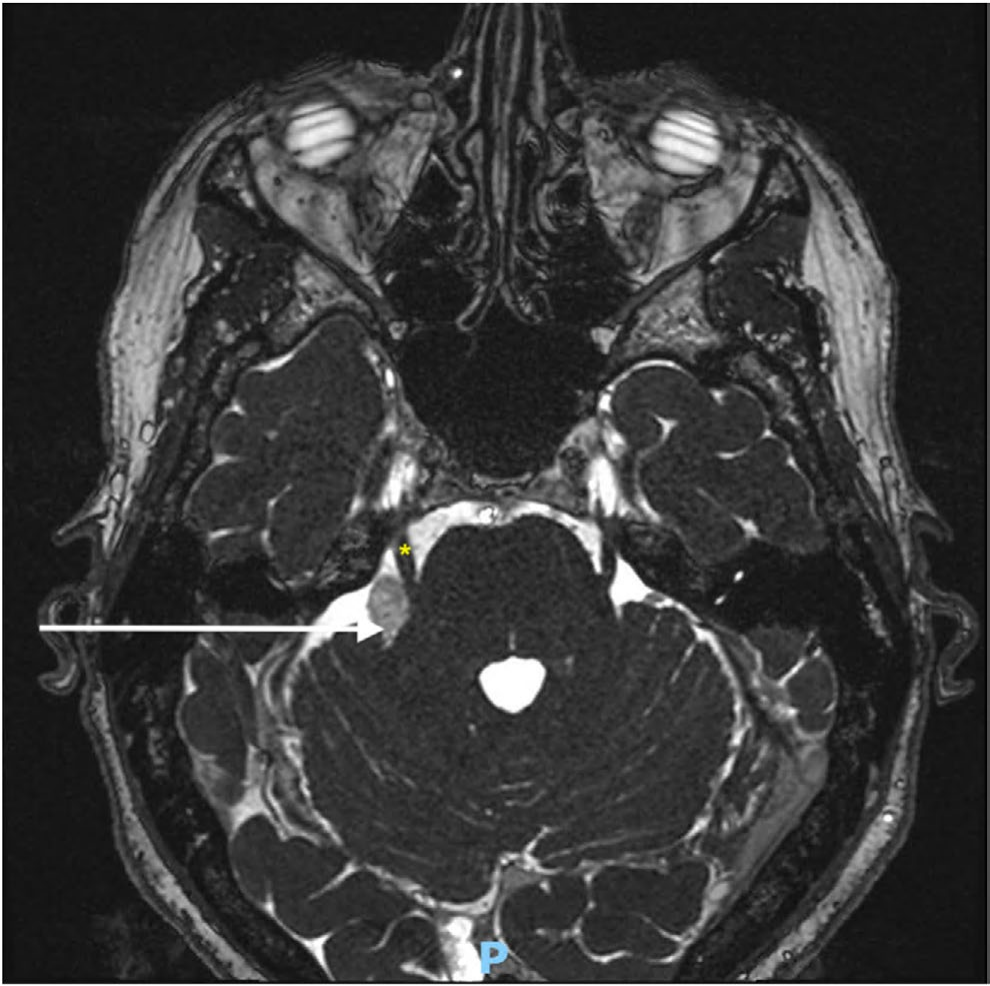
Preoperative MRI (T2‐weighted sequence) showing the vestibular swhannoma (white arrow) and its proximity with trigeminal nerve (yellow asterisk).

A 58‐year‐old woman was diagnosed with a right vestibular schwannoma after experiencing unilateral sensorineural hearing loss. No other symptoms were present. Her medical history included active tobacco use and depressive disorder. Brain magnetic resonance imaging (MRI) with gadolinium revealed a stage III vestibular schwannoma according to Koos classification (Figure 1), in proximity to the trigeminal nerve (Figure 2). This classification is primarily used to evaluate cerebellopontine angle tumors and is based on the tumor's size and its degree of impact on surrounding structures, particularly the brain stem. Following a multidisciplinary discussion involving otolaryngologists, neurosurgeons, radiation oncologists, and medical oncologists, a decision was made to proceed with surgical resection (radiotherapy was not selected due to the proximity of the tumor to the trigeminal nerve). The procedure involved a translabyrinthine resection by a dual team consisting of an otolaryngologist and a neurosurgeon. The surgery lasted 9 hours, with complete resection achieved and no adverse events during the operation.

Upon awakening, the patient exhibited normal clinical findings except for grade II peripheral facial paralysis (PFP) (as per the House–Brackmann scale), along with right‐sided nystagmus and some dizziness. She spent one night in the intensive care unit before returning to the ENT ward. The postoperative protocol included antibiotic prophylaxis with cefazolin 1 g every 8 h for 48 h, acetazolamide 500 mg/day for 48 h to reduce intracranial pressure, and laxatives also to reduce intracranial pressure (by avoiding closed glottis straining).

## 3 Methods

**FIGURE 3:**
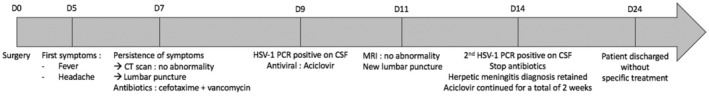
Timeline of events. D: Day; HSV: herpes simplex virus; MRI: magnetic brain resonance imaging; PCR: polymerase chain reaction; CSF: cerebrospinal fluid.

Postoperative days 1 to 4 proceeded without incident, with only moderate headaches and balance disturbances. On day 5, she developed severe headaches with a fever of 38.3°C; neurological examination was normal except for nystagmus and PFP, and examination of the scar showed no signs of local infection. Infection was investigated through chest X‐ray, urine analysis, and blood cultures, all of which returned normal results. Blood tests revealed a biological inflammatory syndrome (lymphocytes at 11000 /mm^3^ and CRP at 32 mg/L). On day 7, the patient continued to have a persistent fever at 38.6°C with a slight increase in the inflammatory syndrome. A brain CT scan then showed no signs of abscess or venous thrombosis. A lumbar puncture was performed on the same day, showing increased lymphocytic pleocytosis and decreased glucose levels suggestive of meningitis, but no bacteria were observed on direct examination (Table 1). Empirical antibiotic therapy with IV cefotaxime (3 g x 4/day) combined with vancomycin (1 g x 2/day) was initiated following consultation with the infectious disease specialist. Viral PCR testing of the CSF returned positive for HSV‐1 and negative for other viruses (HSV‐2, CMV, enterovirus, HHV‐6, parechovirus, VZV). Intravenous acyclovir was therefore added (10 mg/kg x 3/day) for suspected HSVE. The following day, fever persisted (38.2°C), and the patient had a partial epileptic seizure; an EEG was performed and was normal. On day 11, a repeat lumbar puncture with a new HSV‐1 PCR confirmed the HSV‐1 infection, and a brain MRI showed no signs of brain lesions. An afebrile state was eventually achieved, antibiotics were discontinued as no bacteria were observed on CSF examination, and the diagnosis of HSVE was established; a repeat EEG was normal. A timeline is shown in Figure 3.

**TABLE 1:** Lumbar puncture results.

	First lumbar puncture (Day 7)	Second lumbar puncture (Day 11)	Standards
Erythrocytes (/mm^3^)	4230	> 10,000	< 5
Lymphocytes (/mm^3^)	50	166	< 5
Lactate (mg/L)	673	500	108–189
Proteins (g/L)	2.09	1.73	0.15–0.45
Glucose (g/L)	0.23	0.20	2/3 of blood glucose

## 4 Conclusion and Results

On day 24, the patient was discharged after 2 weeks of IV acyclovir, with grade II PFP and some balance disturbances. She apparently had no sequelae from HSVE, particularly no epileptic seizures. A follow‐up consultation at 2 months showed mild PFP (grade II) and balance disturbances without dizziness; no sequelae from HSVE were present.

This case report should draw practitioners’ attention to the fact that after surgery involving cranial nerves, especially cranial nerves VII and VIII due to their proximity to cranial nerve V, and more generally the central nervous system; non‐bacterial meningitis should raise suspicion of herpes simplex virus type 1 (HSV‐1) reactivation leading to HSVE. No time should be wasted in diagnosis and treatment; therefore, a PCR testing on CSF should be systematic in this situation, without neglecting to consider other differential diagnoses.

## 5 Discussion

The herpes simplex viruses are human neurotropic viruses typically acquired during childhood. They remain latent in sensory nerves, including the dorsal roots and cranial nerves [4]. Reactivation of these viruses can occur throughout life, leading to conditions such as herpes labialis, herpetic keratitis, and, in severe cases, meningoencephalitis. HSVE commonly presents with necrosis in the frontotemporal regions of the brain, likely due to the virus's entry via the olfactory route [9]. Another pathway for HSV‐1 entry into the central nervous system is through the cornea, with the virus spreading to the trigeminal ganglion [10]. The trigeminal ganglion is a key site of HSV‐1 latency, and manipulation of this nerve during surgery may trigger viral reactivation. However, this is speculative, and further research is necessary as other hypotheses have been proposed [7]. Additionally, cases of HSVE following neurosurgery have been reported, with epilepsy surgeries more frequently involved, likely due to manipulation of the temporal lobe [11, 12]. In our case, HSV‐1 PCR was ordered upon the recommendation of infectious disease specialists, whose systematic approach to differential diagnosis helped ensure we did not miss the diagnosis. Other possibilities, such as aseptic meningitis (common after neurosurgery [13]), decapitated bacterial meningitis, or drug‐induced meningitis, were also considered [14].

## Author Contributions

**Jérôme Houdu:** conceptualization, data curation, validation, writing – original draft, writing – review and editing. **Maxime Barron:** supervision, validation, visualization. **Thierry Civit:** supervision, validation, visualization. **Cécile Parietti‐Winkler:** supervision, validation, visualization.

## Disclosure

The authors declare that they have no conflict of interest.

## Ethics Statement

Complete written informed consent was obtained from the patient for the publication of this study and accompanying images.

## Data Availability

Data sharing is not applicable to this article as no new data were created or analyzed in this study.
